# Reliability and validity of the interRAI Check-Up Self-Reported (CUSR) Assessment

**DOI:** 10.1186/s12877-025-06021-5

**Published:** 2025-07-02

**Authors:** Hongsoo Kim, Young-il Jung, Seungyeon Chun

**Affiliations:** 1 Department of Public Health Sciences, Graduate School of Public Health, Institute of Health and Environment, and Artificial Intelligence Institute, Seoul National University, Seoul, South Korea; 2 Department of Environmental Health, Korea National Open University, Jongno-Gu, Seoul, South Korea; 3 Department of Public Health Sciences, Graduate School of Public Health, Seoul National University, Seoul, South Korea

**Keywords:** interRAI CUSR, Self-assessment, Functional status, Community care, Older adults, Psychometric evaluation

## Abstract

**Background:**

As the global population ages, the need for community-based tools to assess the functional status and care needs of older adults is increasing. This study examined the reliability, validity, and feasibility of the interRAI Check-Up Self-Reported (CUSR) in a low-income urban district in Seoul, South Korea.

**Methods:**

A total of 158 older adults participated in this study. The interRAI CUSR was administered by trained lay interviewers, and psychometric testing was conducted using interrater reliability, test–retest reliability, and criterion validity measures. The study assessed the agreement between self-reported and clinician-administered assessments as well as participant feedback on feasibility and acceptability.

**Results:**

The interRAI CUSR demonstrated good psychometric properties, with interrater reliability scores (mean kappa = 0.97, Gwet’s AC1 = 0.99) and test–retest reliability (mean kappa = 0.76, Gwet’s AC1 = 0.94), indicating high levels of agreement. Criterion validity showed strong correlations with the items in the interRAI Check-Up (CU) administered by clinicians. The participants reported that the tool was easy to use and accurately reflected their health status. Overall, the interRAI CUSR was found to be a reliable and feasible tool for generally healthy, pre-frail older adults residing in low-income communities.

**Conclusion:**

The interRAI CUSR is a valuable tool for assessing the functional status of older adults living independently in low-income communities. By enabling self-assessment with the support of laypersons, this tool may reduce the burden on health care professionals and enhance community-based care. Further research is required to expand its applicability to diverse settings and populations.

## Background

Healthy aging is a global health policy agenda because the populations of all countries are aging [1, 2]. The coronavirus disease (COVID-19) pandemic has underscored the importance of building healthcare systems that maximize the functional abilities of older adults. This is particularly crucial for social health protection as high mortality rates and physical and mental challenges disproportionately affect older populations [3]. Reliable health surveillance data, including minimum functional profiles for relatively healthy older people in communities, can be invaluable for developing health and well-being programs that aim to prevent and delay the institutionalization of long-term care, one of the most important public health goals in the post-COVID-19 era [4, 5].

Establishing a community-wide function-focused surveillance system is the cornerstone of a well-functioning primary care system. Traditionally, these systems require well-trained professional assessors, typically registered nurses. However, during and after the COVID-19 pandemic, a shortage of professional primary healthcare workers has become prevalent. Consequently, service delivery must become more effective and efficient to reduce the workload of these professionals. On the other hand, empowering and engaging older adults and community laypersons (e.g., friends, family, or neighbors) in primary care services by improving their health literacy and self-care competency is key to the successful implementation and sustainability of community-based healthy aging programs [6, 7].

The value of comprehensive geriatric assessments (CGAs) for individuals with home health and institutional care needs is well-documented. However, there is limited evidence on multidimensional functional assessments for relatively healthy community-dwelling older adults who can assess their own functional status, either independently or with the help of laypersons. Implementing such screening-type CGAs for people with light care needs and focusing more on health and wellness promotion for community-dwelling older adults, with the involvement of community members, would be a good starting point for a function-based surveillance system. This system can identify and refer at-risk groups to appropriate professionals in advance. Such a community-driven, professionally supported health promotion approach can be sustainable when the professional workforce is limited. Moreover, it may be an effective way of empowering communities.

The missing element needed to build a foundation for an innovative ecosystem for healthy aging is psychometrically sound instruments. Recently, the interRAI Check-Up Self-Reported (CUSR) has emerged as a potential option to fill this evidence gap [8, 9]. The interRAI CUSR is a 90-item assessment tool newly added to the interRAI family of health assessment systems and comprises 24 comprehensive and integrated assessment tools. The interRAI CUSR is a self-reported version of the interRAI Check-Up (CU). The reliability of the interRAI CUSR has been tested among community-dwelling older adults in Cape Town, South Africa, a resource-limited setting [10]. A more recent study [11] reported its psychometric properties, feasibility, and usability in home care agencies in Ontario, Canada, a high-income country with different health and long-term care systems and resources, and the results are promising. With its good psychometric properties, the interRAI CUSR is reported to be a reliable and feasible assessment tool that can be used in primary care, community services, and patient-reported outcome measurement research with the general population.

This international evidence motivates the present assessment of the psychometric properties of the interRAI CUSR among older adults living in urban, low-income communities in an Asian context. Seoul, the capital of South Korea, is a megacity of approximately 9.9 million citizens, of which approximately 15.8% were aged 65 years or older in 2020, and this number is expected to rise to 20.1% by 2025 [12]. Healthy aging is a key priority for Seoul, which has actively worked to increase access to and quality of health promotion and preventive health care for community-dwelling older adults. This study assessed the reliability and validity of the interRAI CUSR in a cultural and healthcare system context that differs from those found in the existing literature.

## Methods

### Study setting and sample

This study was conducted in a district in Seoul, South Korea, where the average income level was the third lowest among 25 districts in Seoul in 2019. Using the K-Frailty screening tool [13], independently living community-dwelling older adults classified as generally healthy or pre-frail were identified as the study population. We began our recruitment with older people who had participated in health disparity intervention studies and other programs in community service centers. Voluntary participants were recruited by posting research recruitment brochures on bulletin boards at public health centers and community service centers and operating a research recruitment booth. Participants were older local residents who voluntarily agreed to participate after reading the research recruitment brochure. Those interested in participating in the study were contacted using contact information from the published recruitment document or provided with their own contact information at the research recruitment booth. Consent to participate was obtained from the researcher before starting the inter-RAI CUSR assessment. The target number of research participants was selected by referring to previous studies [10, 11] similar to the present study. A total of 158 older adults provided written informed consent to participate in the study. The study was approved by the institutional review board of the institution to which the first author was affiliated.

### Instrument: interRAI CUSR

The interRAI CUSR [14] is an approximately 90-item functional assessment instrument that covers key aspects of the health and well-being of community-dwelling individuals. Although it does not specifically target older adults, it is suitable for older adults who are likely to have hidden and/or potential risks of functional decline. The instrument includes several subsections covering service use as well as physical, mental, and social functions, including activities of daily living (ADL), instrumental activities of daily living (IADL), cognition, social relationships, health behaviors (lifestyle), health symptoms, and health service utilization. Thus, it is suitable for older adults who are likely to have hidden and/or potential risks for functional decline. The interRAI CUSR is a self-reported version of the interRAI Check-Up (CU), which targets healthier community populations. The interRAI CU items are derived from the interRAI Home Care (HC) assessment and are part of the interRAI health assessment system, a collection of approximately 20 harmonized, comprehensive functional assessment tools that consist of valid and reliable common core items as well as items specific to various care settings and for various populations with special care needs. The widespread use of interRAI assessment systems for clinical, administrative, and research purposes across countries has been reported elsewhere [15]. 

The Korean version of the interRAI HC has been developed and psychometrically tested [16], and the majority of the interRAI CU items are shared with the interRAI HC. In developing the Korean version of the interRAI CU and CUSR, newly added CU items as well as those unique to the interRAI CUSR were translated and back-translated using the same approach as that used in the development of the Korean interRAI HC, as explained in detail by Kim et al. [16]. The instrument was refined through an iterative development process, incorporating reviews and comments from experts, nurses, and laypeople.

### Reliability and validity testing

We implemented interRAI CUSR through interviews with trained community laypeople using an app developed for research purposes. Initially, we attempted to implement the instrument as a truly self-reported version during pilot tests but found it unlikely to yield reliable answers in a relatively low-income, low-education older population. Particularly, assessing their own functions using a standardized tool was an unusual experience for our participants, many of whom had never done so before. The interviewers were middle- and older-aged citizens living in the same neighborhood. They were recruited through public health centers and were interested in serving their communities. The interviewers attended a two-day training course that included guidance on the assessment procedure, lectures by experts on the evaluation method for each question, and instructions on how to use the application for the survey. Assessment exercises were then conducted using practice cases, and the interviewers were instructed to record the responses as older adults reported them without using their own observations. The interRAI CUSR was administered to each participant in an interview format by a trained lay interviewer. The CUSR app was loaded onto the tablet PCs used in this psychometric testing study.

We assessed the psychometric properties of the tool in three ways: inter-rater reliability between two lay interviewers (Test 1), test–retest reliability with a single trained lay interviewer (Test 2), and criterion validity by comparing the interRAI CUSR assessment with the interRAI CU, a clinical assessor version of the interRAI CUSR administered by a registered nurse (Test 3). For reliability testing, the interRAI CUSR was completed by 158 older adults in an interview format with a trained lay interviewer at the participants’ homes or community wellness centers. Another lay interviewer attended the interview session and rated the inter-RAI CUSR concurrently but independently. A total of 93 dyads were created to assess the inter-rater reliability. To assess the test–retest reliability, the second interRAI CUSR (Time 2) was completed by the same 158 older adults who completed the first interRAI CUSR (Time 1). The two self-assessments, with the assistance of a lay interviewer, were conducted within 48 h to minimize the possibility of changes in older participants’ conditions, similar to a previous study [10].

Criterion validity was evaluated. The interRAI CU was administered by a trained registered nurse within 48 h after the interRAI CUSR was completed through an interview with laypersons. The time window for the retest was the same as that used in a previous study [10]. Laypersons assisting older adults in completing the inter-RAI CUSR were instructed not to use their own observations or judgments when recording their reports. By contrast, clinical assessors were guided to use their clinical knowledge and experience, assess older adults using all possible sources of information, and make their own clinical judgments. In total, 158 dyads were collected from the inter-RAI CUSR and CU assessments.

### Data analysis

To check the reliability of the interRAI CUSR, inter-rater reliability was examined using Time 1 interRAI CUSR data, and test–retest reliability was assessed by comparing Time 1 and Time 2 interRAI CUSR data. The proportion of exact agreement (percent agreement), Cohen’s kappa statistics, and the intraclass correlation coefficient (ICC) were computed to assess the overall agreement of the items in the interRAI CUSR. We also computed Gwet’s AC1, as some items suffered from the kappa paradox, where low kappa values are due to low prevalence and marginal probability (e.g., very low kappa values despite a high proportion of actual agreement) [17, 18]. Gwet’s AC1 is a first-order agreement coefficient that makes overall adjustments based on the probability that the raters may agree on a specific rating.

To check the criterion validity of the interRAI CUSR, percent agreement, Cohen’s kappa, Gwet’s AC1, and ICC were also computed to examine the agreement between the items in the interRAI CUSR collected by trained lay interviewers and the same items in the interRAI CU collected by clinical assessors, as the gold standard. The kappa values were evaluated following Landis and Koch: slight (0 to 0.20), fair (0.21 to 0.40), moderate (0.41 to 0.60), substantial (0.61 to 0.80), and almost perfect (0.81 to 1.0) [19]. Gwet’s AC1 applies Landis and Koch’s criteria in the same manner as kappa [18, 20]. An ICC value of 0.90 or higher was interpreted as excellent reliability, 0.75 to 0.90 as good reliability, 0.50 to 0.75 as moderate reliability, and less than 0.50 as low reliability [21]. Statistical analyses were performed using SAS version 9.4.

In addition, similar to the study by Iheme et al. [11], the feasibility and acceptability were assessed by measuring the time taken to complete the assessment, gathering direct feedback about difficult items, and participants’ thoughts about conducting a self-functional assessment. User experience was also evaluated using a structured questionnaire after all psychometric tests were completed. The questionnaire included questions about the difficulty of completing the self-report tool, identifying any questions that were challenging to answer, whether any questions were embarrassment or offensive, how well the survey addressed the respondents’ health needs, any important health concerns not covered by the survey, and any questions that the respondent would choose to remove.

## Results

### Participant characteristics

The participants’ profiles are summarized in Table 1. A total of 158 older adults participated in the study (Tests 2 and 3), of whom 93 also participated in Test 1. The mean age of the participants was 73.9 years. Of the participants, 69.0% were female, 57.6% were married, and more than one-third (34.8%) of the participants lived alone. The average number of underlying diseases was 1.2, with coronary heart disease being the most common (62.7%), followed by diabetes (31.7%), cancer (18.4%), and stroke (5.1%).

**Table 1:** General characteristics of the participants

		Inter-rater reliability (Test 1)		Test-retest reliability & Criterion validity (Tests 2 & 3)	
Sample		*n*=93		*n*=158	
		n	%	n	%
Sex	Male	23	24.7	49	31.0
	Female	70	75.3	109	69.0
Age	Mean, SD	74.2	4.8	73.9	5.0
	60–74	47	50.5	83	52.5
	75+	46	49.5	75	47.5
Marital status	Married	41	44.1	91	57.6
	Widowed, never married, separated, etc.	52	55.9	67	42.4
Living arrangements	Alone	46	49.5	55	34.8
	With spouse	30	32.3	64	40.5
	With child with/without siblings	17	18.3	39	24.7
Underlying diseases	Mean, SD	1.2	0.85	1.2	0.9
	0	19	20.4	35	22.2
	1	42	45.2	69	43.7
	2	27	29.0	43	27.2
	3+	5	5.4	11	7.0
	Coronary heart disease	58	62.4	99	62.7
	Diabetes	33	35.5	50	31.7
	Cancer	17	18.3	29	18.4
	Stroke	4	4.3	8	5.1
Self-reported health	Excellent	3	3.2	9	5.7
	Good	29	31.2	54	34.2
	Fair	53	57.0	80	50.6
	Poor	8	8.6	15	9.5

### Reliability

The inter-rater reliability of the interRAI CUSR was examined to evaluate whether the self-report instrument for older adults was consistently administered by trained laypersons without healthcare expertise (Test 1, Table 2). The grand mean kappa and Gwet’s AC1 statistic for inter-rater reliability were 0.97 and 0.99, respectively. The average kappa statistics for all the tested sections were greater than 0.90. According to Landis and Koch’s criterion, the kappa statistics of the 77 items tested for inter-rater reliability were almost perfect for 89.6% of the items, substantial for 6.5%, moderate and fair for 0%, and 3.9% (3 items) were not calculated due to distribution issues (Appendix 1). The average simple agreement was high (99.2%). The average ICC for continuous and ordinal variables was 0.98, ranging from 0.79 to 1.00, with most items showing excellent reliability.

**Table 2:** Average inter-rater reliability measures of the interRAI CUSR (Test 1)

Section	Number of Items	Simple agreement (%)	Kappa value	Gwet’s AC1	Intraclass correlation coefficient
A. Identification Information	3	99.6	1.00	1.00	
B. Thinking and Communication	6	99.5	0.93	0.99	0.95
C. Well-Being	9	96.5	0.93	0.96	1.00
D. Daily Activities	23	99.9	0.99*	1.00	0.99
E. Health Conditions	23	99.1	0.97	0.99	0.97
F. Disease Diagnoses	7	99.5	0.99*	0.99	
G. Nutrition	3	98.6	0.93*	0.99	
H. Procedures/Treatments	4	100.0	1.00	1.00	1.00
I. Finances and Stressor	2	99.5	0.98	0.99	
Grand mean	80	99.2	0.97	0.99	0.98

^*^For each of these kappa values, one item was omitted because of very low prevalence and highly skewed distribution due to high proportion of actual agreement

Test–retest reliability of the self-assessment instrument for community-dwelling older adults was administered by a layperson within 48 h of the first test (Test 2; Table 3). The grand mean of the kappa and Gwet’s AC1 statistic for test–retest reliability were 0.76 and 0.94, respectively. The kappa statistics of most tested items were 0.4 or above, except for four items (change in decision-making, vision, unstable conditions, and one or fewer meals per day; Appendix 2). Gwet’s AC1 statistics for all tested items were above 0.4. According to Landis and Koch’s criterion, the kappa statistics of the 77 items tested for test–retest reliability were almost perfect for 45.5% of the items, substantial for 24.7%, moderate for 20.8%, fair for 6.5%, and not calculated for 2.6% (2 items). The average agreement was high (94.8%). The mean agreement statistics for the tested CUSR single or group of items were all above 70%, except for the “total hours of exercise or physical activity” item (55.8%; Appendix 2). The average ICC for continuous and ordinal variables was 0.71, with most items showing moderate-to-good reliability.

**Table 3:** Average test–retest reliability measures of the interRAI CUSR (Test 2)

Section	Number of Items	Simple agreement (%)	Kappa value	Gwet’s AC1	Intraclass correlation coefficient
A. Identification Information	3	100.0	1.00	1.00	
B. Thinking and Communication	6	96.9	0.52	0.97	0.60
C. Well-Being	9	89.8	0.67	0.88	0.75
D. Daily Activities	23	97.2	0.88*	0.86	0.62
E. Health Conditions	23	90.8	0.68	0.89	0.75
F. Disease Diagnoses	7	98.2	0.95	0.98	
G. Nutrition	3	98.4	0.62	0.98	
H. Procedures/Treatments	4	99.4	0.96	0.99	0.63
I. Finances and Stressors	2	95.4	0.61	0.94	
Grand mean	80	94.8	0.76	0.94	0.71

^*^The value is based on the average kappa of twenty-one items, as the kappa for two items could not be computed owing to very low prevalence and highly skewed distribution due to high proportion of simple agreement

### Validity

The grand mean kappa statistic of all tested interRAI CUSR items for criterion validity was 0.88 (Table 4, Test 3). The mean kappa statistics across the eight tested sections were all greater than 0.80, except for the Thinking and Communication section (0.64), where the average agreement ranged from 94.9% to 99.8% (highly skewed distribution). The kappa statistics of all tested items for validity were above 0.60, except for the “change in decision-making” (0.49) and “making self-understood” (0.57) items (Appendix 3). None of the items had kappa values below 0.40. Among the items, 2.6% had kappa values ranging between 0.41 and 0.60, 24.7% had kappa values between 0.61 and 0.80, and 71.4% had values between 0.81 and 1.00. The lowest percent-agreement statistic for validity was observed for the “total hours of exercise or physical activity” item (85.4%; Appendix 3). The ICC for continuous and ordinal variables was above 0.6 for all items. The average ICC score for each section ranged from 0.77 (Thinking and Communication) to 0.91 (Health Conditions).

**Table 4:** Average criterion validity of paired items in the interRAI CUSR and interRAI CU (Test 3)

Section	Number of Items	Simple agreement (%)	Kappa value	Gwet’s AC1	Intraclass correlation coefficient
A. Identification Information	3	99.8	1.00	1.00	
B. Thinking and Communication	6	97.5	0.64	0.97	0.77
C. Well-Being	9	94.9	0.84	0.94	0.89
D. Daily Activities	23	99.0	0.95*	0.99	0.90
E. Health Conditions	23	96.2	0.87	0.95	0.91
F. Disease Diagnoses	7	97.3	0.90	0.97	
G. Nutrition	3	99.2	0.85	0.99	
H. Procedures/Treatments	4	97.8	0.88	0.97	0.85
I. Finances and Stressors	2	98.5	0.86	0.98	
Grand mean	80	97.4	0.88	0.97	0.88

^*^The value is based on the average kappa of twenty-two items, as the kappa for one item could not be computed owing to its very low prevalence and highly skewed distribution due to high proportion of simple agreement

### Feasibility

A total of 104 participants (lay interviews) responded to questions about their user experience to assess the usability of the tool. Most participants (99%) reported that the questions were not difficult, and 99% found the questions satisfactory, with none of the questions embarrassing or unpleasant. In response to the question of how well the evaluation tool identified their health status, participants generally felt that it accurately reflected their health. Overall, the tool was perceived as applicable to older adults living in Korean communities.

## Discussion

As building a people-centered, integrated healthcare system is the direction for future health systems, collecting people-reported functional status and care needs is essential [7, 22]. This study suggests that the interRAI CUSR conducted by trained community laypersons can be a useful tool for achieving this, particularly among lower socioeconomic communities with lower (digital) health literacy during the COVID-19 pandemic, as they can complete their self-assessment with the assistance of trained laypersons. The study also highlights that the interRAI CUSR can reduce the assessment burden on community nurses and that it is relatively easy for older persons to recruit volunteers as lay interviewers to complete the interRAI CUSR. Furthermore, the tool may facilitate the empowerment and engagement of neighbors in the community to provide peer support for elderly care.

The findings of this study align with those of previous studies that tested the psychometric properties of the interRAI CUSR in other countries and suggest that the tool can be applied to various care settings and populations. For example, Geffen et al. [10] reported that interRAI CUSR showed good reliability and feasibility in South Africa, a low-resource setting. Similarly, Iheme et al. [11] found that the tool had strong psychometric properties and was suitable for use in home care agencies in Ontario, Canada. These findings confirm that the tool can be used in various cultural and healthcare contexts, including urban and low-income community settings in Korea.

One of the strengths of the interRAI CUSR is that it is a part of the interRAI suite of assessment systems, which allows for the integration of self-reported data with other interRAI instruments used in clinical and long-term care settings. Building on this, following a previous study in Korea that validated the interRAI long term care facilities (LTCF) and home care (HC) for frail older people [16], this study confirmed the suitability of the interRAI CUSR for healthy community-dwelling older adults, making it a valuable continuous information system. This is particularly important because it provides a more comprehensive view of the functional status and care needs of older persons. Moreover, the use of self-report assessments can empower older adults by giving them an active role in monitoring their health and making decisions regarding care. The high level of agreement between the self-reported, lay interviewer-assisted, and clinician-administered assessments found in this study suggests that CUSR is a reliable tool for self-assessment without the help of a healthcare provider, even in populations with lower health literacy. The data collected can be used not only for profiling but also for patient-reported outcome measures (PROMs) to evaluate service effectiveness. The interRAI CUSR aims to change from the traditional interRAI CGA system, in which clinicians evaluate older adults’ comprehensive conditions, to a self-report method by older adults. It is often a challenge for older adults to comprehensively understand their own health. interRAI researchers’ extensive research experience has been complemented by a system that enables older adults to self-report their functional health status, providing a range of PROMs (e.g., physical function, cognition, social relationships, and mood). This information highlights areas that require intervention or improvement and can also be used to evaluate the effectiveness of services, as changes can be identified through repeated measurements. Previous efforts to implement PROMs have been hindered by factors such as frailty and low literacy [23, 24]; however, the interRAI CUSR may offer a promising alternative.

This study has some limitations. First, the sample was drawn from a single urban district in Seoul, which may limit the generalizability of the findings to other settings, particularly rural areas or regions with different socioeconomic conditions. In addition, in the context of the COVID-19 pandemic, frail older adults were excluded for safety reasons. Second, although the interRAI CUSR was administered with the help of laypersons, some participants with low literacy may still have found certain questions difficult to answer accurately in assessing their own functional abilities. Future research should consider expanding the sample to include more diverse populations and settings, as well as exploring strategies to further simplify the assessment process for older adults with lower health literacy.

## Conclusion

This study demonstrated that the interRAI CUSR is a valuable tool for assessing the functional status of generally healthy and pre-frail older adults, particularly in lower socioeconomic and health literacy settings. By enabling self-assessment with layperson assistance, it might empower older adults and reduce the burden on healthcare professionals, thereby supporting the development of people-centered, integrated care systems. However, to maximize its applicability, further research should focus on expanding its use to different settings and populations, as well as simplifying the assessment process for individuals with lower health literacy. Ultimately, the interRAI CUSR contributes to the development of more inclusive and efficient health and care systems, supporting the goal of healthy aging in diverse community contexts.

## Data Availability

The datasets generated and/or analyzed in the current study are not publicly available because of the SNU IRB policy, which prohibits the sharing of research data with any third party. The data are available from the corresponding author upon request.

## **Appendix 1 Inter-rater reliability measures of items in the interRAI (Test 1)**

**Table:**

Section	Number of Items	Simple agreement (%)	Kappa value	Gwet's AC1	Intraclass correlation coefficient
**A. Identification Information **	3				
Sex	1	100.0	1.00	1.00	**-**
Marital status	1	100.0	1.00	1.00	**-**
Living arrangements	1	98.9	0.99	1.00	**-**
**B. Thinking and Communication**	6				
Decisions about daily tasks	1	100.0	1.00	1.00	1.00
Change in decision-making	1	98.9	0.80	0.99	-
Making self understood	1	100.0	1.00	1.00	1.00
Ability to understand others	1	100.0	1.00	1.00	1.00
Vision	1	97.8	0.79	0.98	0.79
Hearing	1	100.0	1.00	1.00	1.00
**C. Well-Being**	9				
Mood	3	100.0	1.00	1.00	1.00
Social activities	1	92.5	0.91	0.92	-
Degree of loneliness	1	98.9	0.98	0.99	0.99
Close friend in community	1	97.8	0.94	0.97	-
Hours of informal care (Continuous variable)	1	.	-	-	1.00
Family overwhelmed	1	98.9	0.88	0.99	*-*
Change in social activities	1	83.9	0.71	0.79	-
**D. Daily Activities**	23				
IADL	8	99.9	1.00*	1.00	-
ADL	10	100.0	1.00	1.00	-
Primary mode of locomotion	1	100.0	1.00	1.00	-
Hours physical activity	1	98.9	0.98	0.99	0.98
Days went out	1	100.0	1.00	1.00	1.00
Change in ADL status	1	98.9	0.79	0.99	-
Drove car	1	100.0	1.00	1.00	-
**E. Health Conditions**	23				
Bladder continence	1	100.0	1.00	1.00	-
Bowel continence	1	97.8	0.95	0.98	-
Fall	3	99.3	0.88	0.99	0.89
Problem frequency	9	99.0	0.98	0.99	0.99
Dyspnea	1	100.0	1.00	1.00	1.00
Fatigue	1	97.8	0.92	0.98	0.94
Pain	2	98.4	0.98	0.98	0.99
Unstable conditions	2	100.0	1.00	1.00	-
Self-rated health	1	98.9	0.98	0.99	-
Smoking & drinking	2	99.5	0.99	0.99	-
**F. Disease Diagnoses**	7				
Total	7	99.5	0.99*	0.99	-
**G. Nutrition**	3				
Weight loss	1	98.9	0.85	0.99	-
One or fewer meals a day	1	96.8	-*	0.97	-
Decrease in food or fluid	1	100.0	1.00	1.00	-
**H. Procedures/Treatments**	4				
Hospitalization, ER visit (Continuous variable)	2	-	-	-	1.00
Prevention	1	100.0	1.00	1.00	-
Wound care	1	100.0	1.00	1.00	-
**I. Finances and Stressors**	2				
Finances	1	100.0	1.00	1.00	-
Major life stressors	1	98.9	0.96	0.99	-
**Criterion summary ** **(Kappa & Gwet's AC1/ICC)**			n=77 (%)	n=77 (%)	n=30 (%)
Almost perfect/Excellent			89.6	98.7	90.0
Substantial/Good			6.5	1.3	6.7
Moderate/Moderate			0	0	3.3
Fair/Low			0	0	0
Slight			0	0	0
* Uncalculated			3.9	0	0

*ADL* Activities of daily living, *IADL* Instrumental activities of daily living, *ER* Emergency room

*One item’s kappa statistic could not be computed because of its very low prevalence and highly skewed distribution due to high proportion of simple agreement

## **Appendix 2 Test-Retest Reliability Measures of Items in the inteRAI CUSR (Test 2)**

**Table:**

Section	Number of Items	Simple agreement (%)	Kappa value	Gwet's AC1	Intraclass correlation coefficient
**A.** **Identification Information**	3				
Sex	1	100.0	1.00	1.00	-
Marital Status	1	100.0	1.00	1.00	-
Living arrangements	1	100.0	1.00	1.00	-
**B. Thinking and Communication**	6				
Decisions about daily tasks	1	98.8	0.50	0.99	0.50
Change in decision-making	1	95.7	0.35	0.95	-
Making self understood	1	96.2	0.56	0.96	0.61
Ability to understand others	1	99.4	0.80	0.99	0.89
Vision	1	95.7	0.35	0.95	0.35
Hearing	1	95.7	0.58	0.95	0.65
**C. Well-Being**	9				
Mood	3	93.3	0.64	0.93	0.72
Social activities	1	79.1	0.76	0.76	-
Degree of loneliness	1	89.0	0.73	0.88	0.78
Close friend in community	1	94.5	0.85	0.91	
Hours of informal care (Continuous variable)	1	-	-	-	0.84
Family overwhelmed	1	94.5	0.44	0.94	-
Change in social activities	1	81.6	0.63	0.77	-
**D. Daily Activities**	23				
IADL	8	99.5	0.89*	1.00	-
ADL	10	99.9	1.00	1.00	-
Primary mode of locomotion	1	99.4	0.57	0.99	-
Hours physical activity	1	55.8	0.54	0.46	0.67
Days went out	1	90.8	0.56	0.90	0.56
Change in ADL status	1	94.5	0.45	0.94	-
Drove car	1	100.0	1.00	1.00	-
**E. Health Conditions**	23				
Bladder continence	1	89.2	0.78	0.88	-
Bowel continence	1	94.9	0.54	0.94	-
Fall	3	98.1	0.74	0.98	0.77
Problem frequency	9	92.9	0.71	0.92	0.76
Dyspnea	1	94.5	0.62	0.94	0.73
Fatigue	1	86.5	0.57	0.84	0.56
Pain	2	73.7	0.69	0.68	0.77
Unstable conditions	2	88.3	0.39	0.85	-
Self-rated health	1	74.2	0.65	0.68	-
Smoking & drinking	2	97.2	0.92	0.97	-
**F. Disease Diagnoses**	7				
Total	7	98.2	0.95	0.98	-
**G. Nutrition**	3				
Weight loss	1	98.2	0.72	0.98	-
One or fewer meals a day	1	98.2	0.39	0.98	-
Decrease in food or fluid	1	98.8	0.74	0.99	-
**H. Procedures/Treatments**	4				
Hospitalization, ER visit (Continuous variable)	2	-	-	-	0.63
Prevention	1	98.8	0.92	0.99	-
Wound care	1	100.0	1.00	1.00	-
**I. Finances and Stressors**	2				
Finances	1	98.2	0.66	0.98	-
Major life stressors	1	92.6	0.56	0.91	-
**Criterion summary** **(Kappa & Gwet's AC1/ICC)**			n=77 (%)	n=77 (%)	n=30 (%)
Almost perfect/Excellent			45.5	90.9	6.7
Substantial/Good			24.7	7.8	33.3
Moderate/Moderate			20.8	1.3	53.3
Fair/Low			6.5	0	6.7
Slight			0	0	0
* Uncalculated			2.6	0	0

*ADL* Activities of daily living, *IADL* Instrumental activities of daily living, *ER* Emergency room

## **Appendix 3 Criterion Validity of CUSR and CU (Test 3)**

**Table:**

Section	Number of Items	Simple agreement (%)	Kappa value	Gwet's AC1	Intraclass correlation coefficient
**A. Identification Information**	3				
Sex	1	100	1.00	1.00	
Marital Status	1	100	1.00	1.00	
Living arrangements	1	99.4	0.99	0.99	
**B. Thinking and Communication**	6				
Decisions about daily tasks	1	98.1	0.66	0.98	0.90
Change in decision-making	1	97.5	0.49	0.97	
Making self understood	1	95.6	0.57	0.95	0.62
Ability to understand others	1	98.7	0.66	0.99	0.80
Vision	1	98.1	0.79	0.98	0.79
Hearing	1	96.8	0.69	0.97	0.74
**C. Well-Being**	9				
Mood	3	97.7	0.92	0.98	0.96
Social activities	1	91.8	0.85	0.91	
Degree of loneliness	1	92.4	0.86	0.92	0.91
Close friend in community	1	95.6	0.88	0.93	
Hours of informal care (Continuous variable)	1	-	-	-	0.66
Family overwhelmed	1	97.5	0.65	0.97	
Change in social activities	1	88.6	0.75	0.86	
**D. Daily Activities**	23				
IADL	8	99.5	0.92*	1.00	
ADL	10	99.9	1.00	1.00	
Primary mode of locomotion	1	100.0	1.00	1.00	1.00
Hours physical activity	1	85.4	0.84	0.83	0.87
Days went out	1	98.7	0.93	0.98	0.87
Change in ADL status	1	96.8	0.73	0.97	
Drove car	1	99.4	0.97	0.99	
**E. Health Conditions**	23				
Bladder continence	1	91.8	0.70	0.77	
Bowel continence	1	98.7	0.93	0.99	
Fall	3	100.0	1.00	1.00	1.00
Problem Frequency	9	96.3	0.86	0.96	0.90
Dyspnea	1	94.3	0.67	0.94	1.00
Fatigue	1	93.0	0.77	0.92	0.80
Pain	2	92.7	0.91	0.91	0.76
Unstable conditions	2	95.3	0.77	0.94	
Self-rated health	1	93.7	0.91	0.92	
Smoking & drinking	2	99.1	0.99	0.99	
**F. Disease Diagnoses**	7				
Total	7	97.3	0.93	0.97	
**G. Nutrition**	3				
Weight loss	1	98.7	0.79	0.99	
One or fewer meals a day	1	100.0	1.00	1.00	
Decrease in food or fluid	1	98.7	0.74	0.99	
**H. Procedures/Treatments**	4				
Hospitalization, ER visit (Continuous variable)	2				0.85
Prevention	1	95.6	0.75	0.95	
Wound care	1	100.0	1.00	1.00	
**I. Finances and Stressors**	**2**				
Finances	1	98.8	0.83	0.99	
Major life stressors	1	98.2	0.89	0.98	
**Criterion summary ** **(Kappa & Gwet's AC1/ICC)**			n=77 (%)	n=77 (%)	n=30 (%)
Almost perfect/Excellent			71.4	98.7	60.0
Substantial/Good			24.7	1.3	26.7
Moderate/Moderate			2.6	0	13.3
Fair/Low			0	0	0
Slight			0	0	0
* Uncalculated			1.3	0	0

*ADL* Activities of daily living, *IADL* Instrumental activities of daily living, *ER* Emergency room

## Abbreviations

CGA: Comprehensive geriatric assessment
CUSR: Check-up Self-Reported
CU: Check-up
HC: Home care
ICC: Intra-class correlation coefficient
ADL: Activities of daily living
IADL: Instrumental activities of daily living
ER: Emergency room
LTCF: Long term care facilities
